# Short term clinical outcomes and analysis of risk factors for pacemaker implantation: a single center experience of self-expandable TAVI valves

**DOI:** 10.1186/s13019-020-01241-9

**Published:** 2020-07-29

**Authors:** Simon C. Y. Chow, Randolph H. L. Wong, Gary S. H. Cheung, Alex P. W. Lee, Henry K. L. Chui, Kent C. Y. So, Eugene B. Wu

**Affiliations:** 1 Division of Cardiothoracic Surgery, Department of Surgery, The Chinese University of Hong Kong, Prince of Wales Hospital, 30-32 Ngan Shing Street, Shatin, New Territories Hong Kong; 2 Division of Cardiology, Department of Medicine, The Chinese University of Hong Kong, Prince of Wales Hospital, 30-32 Ngan Shing Street, Shatin, New Territories Hong Kong

**Keywords:** Aortic valve stenosis, Transcatheter aortic valve implantation, Cardiac pacemaker, Outcome studies

## Abstract

**Objectives:**

Transcatheter aortic valve implantation is a recognized treatment for patients with severe aortic stenosis at all risk groups. However, permanent pacemaker rates remain high for self expandable transcatheter valves and permanent pacemaker implantation has been associated with increased morbidity. In this analysis we aim to evaluate short term clinical outcomes post self expandable transcatheter aortic valve implantation and determine risk factors for permanent pacemaker implantation.

**Methods:**

88 patients with severe aortic stenosis with transcatheter aortic valve implantation performed between the year 2016–2018 were retrospectively analyzed. Outcomes of interest included 1- year all cause mortality, 30-day major adverse cardiovascular events, permanent pacemaker and paravalvular leak rates. Survival analysis was performed with Kaplan Meier analysis and risk factors for survival and permanent pacemaker rates were identified with log rank test and regression analysis.

**Results:**

The mean age of the cohort was 80.3 +/− 6.9 years. The mean STS score was 9.25. The 30 day all-cause mortality was 5.7% and 1-year all cause mortality was 16.7%. 80 patients had transfemoral transcatheter aortic valve implantation, and a majority of the patients (85.2%) were implanted with Corevalve Evolut R device. The device success rate was 88.6%. Multivariate analysis identified concomitant severe coronary artery disease (OR = 18.2 +/− 0.9; *P* = 0.002), pre transcatheter aortic valve implantation atrial fibrillation (OR = 8.6 +/− 0.91; *P* = 0.02) and post procedural disabling stroke (OR = 32.6 +/− 1.35; *P* = 0.01) as risk factors for 1-year mortality. The 30-day pacemaker rate was 17.6%. The presence of right bundle branch block (OR 11.1 +/− 0.86; *P* = 0.005), non-coronary cusp implantation depth (OR = 1.34 +/− 0.15; *P* = 0.05) and a non coronary cusp implantation depth / membranous septal length ratio of more than 50% were associated with post procedural pacemaker implantation (OR = 29.9 +/− 1.72; *P* = 0.05). Among the 15 patients with post procedural pacemaker implantation, 40% were found to be non-pacemaker dependent at 1 year.

**Conclusion:**

Short term outcomes of transcatheter aortic valve implantation in severe aortic stenosis patients are promising. Pacemaker rates remain high. More studies are needed to evaluate the factors that influence pacemaker rates and dependence to further improve transcatheter aortic valve implantation outcomes.

## Introduction

The emergence of Transcatheter aortic valve implantation (TAVI) has revolutionized the treatment of patients with severe aortic stenosis (AS). The Partner III trial and the Evolut R low risk trial have demonstrated superiority and equipoise of TAVI over surgical aortic valve replacement (SAVR) with respect to short term survival and procedural outcomes, and has firmly established TAVI as an acceptable and effective treatment of severe AS in patients at low risk for SAVR [1, 2]. However, we cannot discount the fact that the aforementioned trials have only included highly selective patients and the majority of the population are elderly with mean age more than 70 years old. The role of TAVI in younger patients is less clear, as important issues such as valve durability,the need for permanent pacemakers (PPM) and bicuspid aortic valves remain to be studied and substantiated [3]. Specifically, the need for PPM post TAVI is particularly important given the added morbidity and negative impact on survival a permanent pacemaker entails to younger patients with life expectancy of more than 10 years. The reported risk of PPM post TAVI range from 10 to 30% [4, 5] irrespective of the type of valve implanted and this rate is much higher than that of SAVR at 3–6% [6]. In our retrospective analysis, we aim to evaluate short term outcomes and efficacy of self-expandable (SE) TAVI valves in our center and identify associating factors that influence PPM rates post TAVI.

## Materials and methods

Between 2016 and 2018, 88 patients underwent TAVI in the Prince of Wales Hospital, Hong Kong. These patients were selected from a local cardiac surgery registry and relevant clinical and radiological data were retrospectively extracted from electronic and paper records for further analysis. All patients were considered not candidates for SAVR by a multidisciplinary HEART team. This cardiac surgical local registry was approved by the Hong Kong Hospital Authority and the Government of Hong Kong SAR to allow collection, analysis, reporting and outcome tracking of patient data since its introduction in 2007. No informed consents from patients were sought for this retrospective analysis as there were no identifiers in this manuscript that could disclose individual patient confidentiality. All patient data had been secured and kept confidential.

The outcomes of interest were consistent with the standardized definitions reported by the VARC 2 consortium [7]. Our results and analysis consisted of evaluation of survival outcomes and major TAVI complications including major vascular complications, para-valvular leak (PVL) and PPM rates. In terms of survival analysis, the outcomes of interest were 30 day and 1 year all cause mortality post TAVI. Procedural related death was defined as deaths within 30 days of TAVI. Cardiovascular mortality was defined as deaths relating to any cardiac or vascular events, which included myocardial infarction, cardiac pump failure, arrhythmias, vascular complications, cerebrovascular disease, sudden or unwitnessed death and death of unknown cause. Procedural related stroke was defined as a modified Rankin (mRS) score of 2 or more at 30 days post TAVI or increase in 1 category from baseline.

**Fig. 1:**
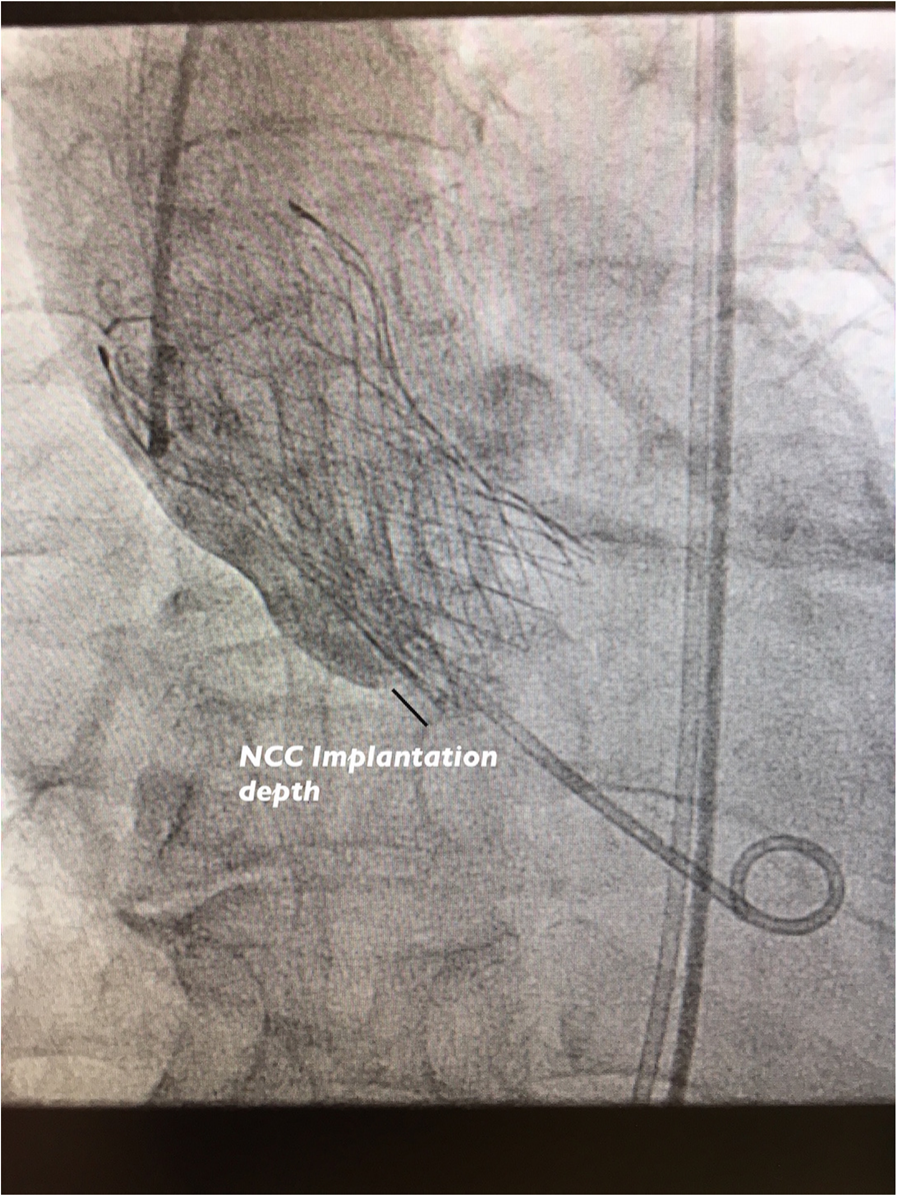
Implantation depth at NCC at implantation angle during deployment (Black line illustrating implantation depth)

**Fig. 2:**
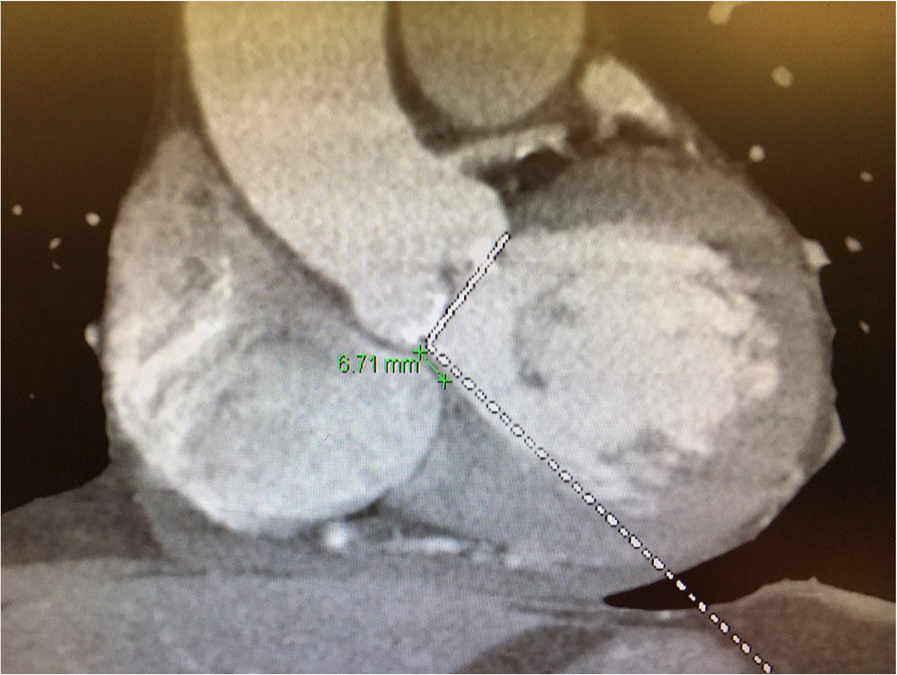
Measurement of infra-annular membranous septal length (IMS) on coronal cut computed tomography images. Distance measured between lowest point of annulus and start of muscular interventricular septum (Green line with measured dimension of IMS length)

Procedural records were reviewed, and peri-operative data were retrospectively recorded and analysed. Important intraoperative data were retrieved which included the infra-annular membranous septal (IMS) length, implantation depth at the non-coronary (NCC) (Fig. 1) and left coronary cusps (LCC) on fluoroscopy. The membranous septal length was measured from pre-TAVI ECG-gated computed tomography images at coronal view with no angulation, as defined as the distance between the lowest margin of the annulus to the start of the interventricular septum (Fig. 2). The ratio between the NCC implantation depth on implantation angle and the IMS length was calculated. Successful device implantation was defined as the absence of procedural related mortality, single valve deployment and the absence of moderate to severe paravalvular leak with a post deployment mean gradient of less than 20 mmHg. The need of PPM was considered as procedural related if an indication for PPM arose within 30 days post TAVI. Pacemaker non dependence was defined as 1) absence of admission for pacemaker related dysfunction or hemodynamically unstable arrhythmias; 2) atrial or ventricular pacing percentage (Vp or Ap) less than 5% during pacemaker interrogation at 1 year post TAVI; 3) baseline follow up electrocardiogram of native rhythm with rate > 60 bpm.

Survival and risk factor analysis was performed using IBM SPSS statistics® Version 25. Continuous variables were presented as mean +/− standard deviation. Categorical variables were expressed as frequencies and percentages. Kaplan Meier analysis was performed for survival analysis. Univariate analysis of peri- operative factors associated with survival and major complications were analysed with the log rank test and binary logistic regression studies. Significant predictors of mortality or major complications had *P* values < 0.05. If more than one factor was found to be associated with an outcome, multivariate analysis was performed to adjust for confounders.

## Results

### Baseline demographics

Table 1 displays the baseline characteristics of the studied population. 88 patients were selected for 30 day analysis and 84 patients had data for 1 year analysis. The mean age of the population was 80.3 +/− 6.9 years. The mean STS predicted risk of mortality score was 9.25. 47.7% patients had moderate to severe ischemic heart disease, in which 34.1% had coronary artery disease severe enough to require revascularisation pre TAVI. 5.7% patients had concomitant moderate to severe valvular disease apart from aortic valve disease. The mean left ventricular ejection fraction (LVEF) was 55.8 +/− 12, and 6.8% patients had a bicuspid aortic valve. 3 patients had pre TAVI pacemaker. Most patients were in sinus rhythm pre TAVI (64.8%), 15.9% patients had right bundle branch block and 15.9% had atrial fibrillation.

**Table 1:** Baseline demographics of patient population.

Baseline (*N* = 88)	
**Age**	80.3 +/− 6.9
**STS score breakdown (N = 88)**	
**Mean STS score**	9.25
**PROM > 8%**	44.3%
**PROM 4–8%**	43.2%
**PROM < 4%**	12.5%
**Pre op Renal replacement therapy**	3/88 (3.4%)
**Presence of Ischemic heart disease**	42/88 (47.7%)
**Need for pre TAVI coronary revascularization (PCI)**	30/88 (34.1%)
**LVEF (*N*** **= 82)**	55.8 +/− 12%
**Presence of other valvular heart disease(moderate or more in severity)**	5/88 (5.7%)
**Bicuspid aortic valve on echocardiogram**	6/88 (6.8%)
**Pre op rhythm**	
**Sinus rhythm**	57/88 (64.8%)
**Atrial fibrillation**	14/88 (15.9%)
**Complete or incomplete bundle branch block**	14/88 (15.9%)
**Permanent pacemaker rhythm**	3/88 (3.4%)

### Procedural characteristics

Table 2 displays the procedural characteristics. 80 patients had transfemoral TAVI, while the rest had trans-aortic TAVI. The majority (85.2%) of TAVI implanted were Corevalve Evolut R (Medtronic Inc., Minneapolis, Minnesota USA), 6.8% of patients had Portico (Abbott Vascular, Abbott Park, IL, USA) valves implanted and 7% of patients had Hydra (Vascular Innovations Co Ltd., Thailand) valves implanted.. 42.5% of the TAVI procedures required pre-dilatation or post dilatation respectively. The mean NCC implantation depth was 4.6 +/− 2.8 mm and the mean LCC implantation depth was 6.4 +/− 3.0 mm. 57 patients had measurements of the IMS length, and the mean IMS length was 9.3 +/− 1.8 mm. 40% patients had an NCC implantation depth and IMS length ratio of more than 0.5.

**Table 2:** Procedural characteristics

*Procedural characteristics*	
*Approach*	
*Transfemoral*	80/88 (90.9%)
*Transaortic*	8/88 (9.1%)
*TAVI valve*	
*EVOLUT R*	75/88 (85.2%)
*PORTICO*	6/88 (6.8%)
*HYDRA*	7 /88 (8%)
*Pre dilatation*	37/87 (42.5%)
*Post dilatation*	37/87 (42.5%)
*NCC depth of implantation (N = 77)*	4.6 +/− 2.8 mm
*LCC depth of implantation (N = 77)*	6.4 +/− 3.0 mm
*Mean infra-annular membranous septal length (n = 57)*	9.3 +/− 1.8 mm
*Implantation depth/IMS length ratio > 0.5*	23/57 (40.4%)

### Survival and outcome analysis

**Fig. 3:**
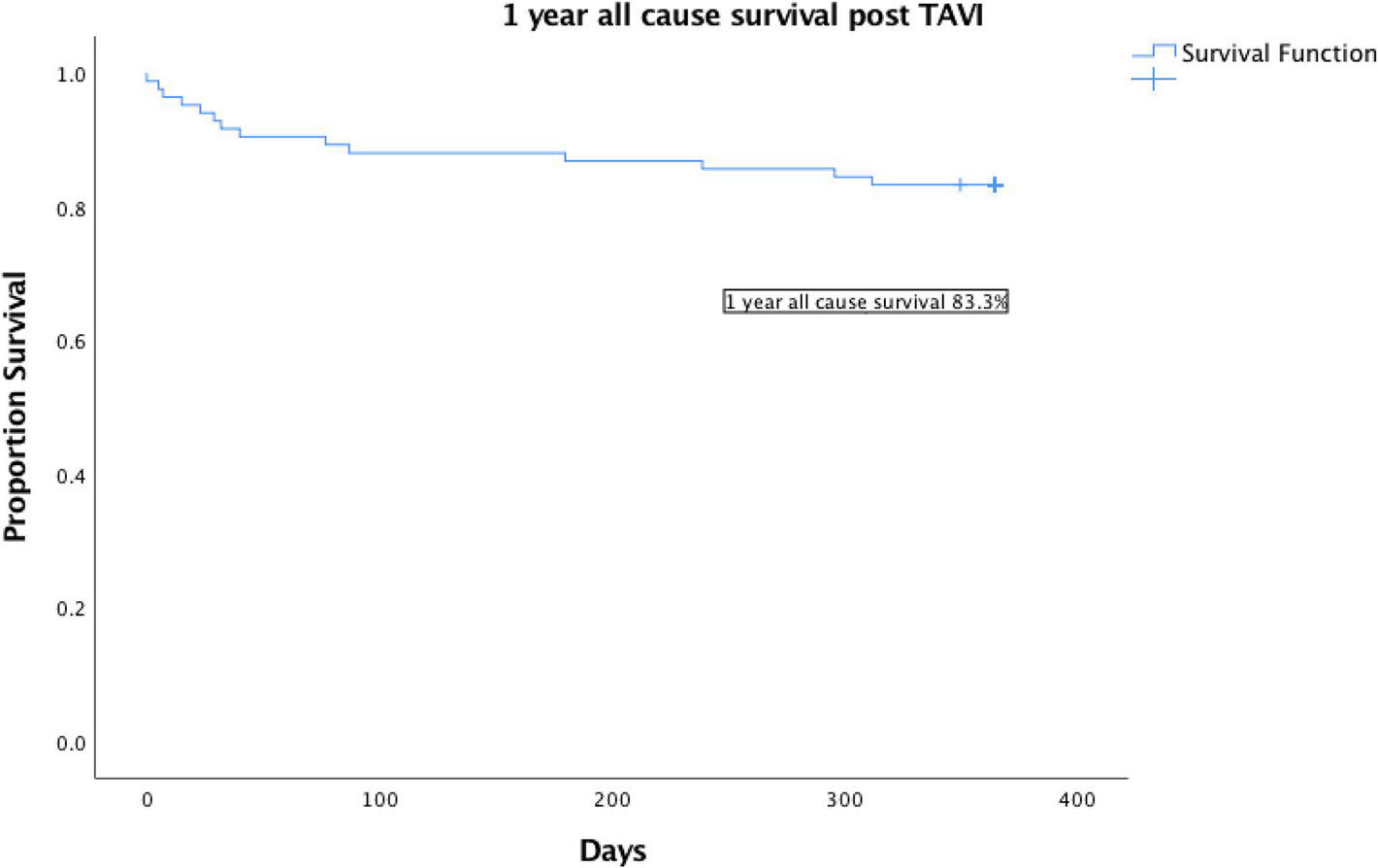
Kaplan Meier analysis of all cause survival post TAVI

Table 3 displays the outcomes post TAVI. The 30 day all cause mortality rate was 5.7%. The 1 year all cause mortality rate by Kaplan Meier analysis was 16.7%, with 1 year all cause survival rate of 83.3% (Fig. 3). The cardiovascular death rate at 1 year was 11.9%. Among the 14 mortalities at 1 year, 3 patients succumbed from disabling stroke, 3 patients had myocardial infarction and 2 patients died from severe heart failure. The commonest cause of death were sepsis (3/14), disabling stroke and myocardial infarction. The rate of major vascular complications was 8%. 2 patients had coronary obstruction post TAVI deployment and 1 case had an intraoperation root rupture. The 30 day disabling stroke rate was 5.7 and 6.8% patients required temporary renal replacement therapy for acute kidney injury. The 30 day PPM rate was 17.6%. The device success rate was 88.6%. At 1 year follow up, 1 patient (1.2%) required repeat valvular intervention and 5/84 (6%) of patients had moderate or severe paravalvular leak on echocardiogram. The mean gradient over the aortic valve was 8.4 +/− 4.4 mmHg at 1 year follow-up echocardiogram. 8.3% of patients had repeated admissions for cardiac events such as angina, myocardial infarction or heart failure at 1 year.

**Table 3:** Outcomes

*Procedural outcomes*	
*30 day all cause mortality*	5/88 (5.7%)
*1 year actuarial all cause mortality*	14/84 (16.7%)
*Kaplan Meier 1 year all cause survival*	83.3%
Causes of death (1 year) * n = 14	
*Sepsis*	3/14 (21.4%)
*Renal failure*	1/14 (7.1%)
*Pacing wire ventricular perforation*	1/14 (7.1%)
*Root rupture*	1/14 (7.1%)
*Myocardial infarction*	3/14 (21.4%)
*Congestive heart failure*	2/14 (14.5%)
*Stroke*	3/14 (21.4%)
*Cardiovascular death at 1 year*	10/84 (11.9%)
*Major Vascular complications*	8/88 (8%)
*Coronary obstruction*	2/88 (2.3%)
*30 day disabling stroke risk*	5/88 (5.7%)
*30 day Acute kidney injury with renal replacement therapy*	6/88 (6.8%)
*Device success rate (defined as single valve,with no moderate to severe PVL, mean gradient < 20 mmHg*)	88.6%
*1 year Prosthetic valve endocarditis*	2/84 (2.4%)
*1 year Repeat valvular intervention*	1/84 (1.2%)
30 Day Pacemaker rates (n = 85 *3 cases of pre op PPM excluded)	15/85 (17.6%)
*Moderate or more paravalvular leak (PVL) on 1 year echocardiogram*	5/84 (6.0%)
*Repeated cardiac events (angina, MI, heart failure) requiring admissions within 1 year*	7/84 (8.3%)

### Risk factor and PPM analysis

**Fig. 4:**
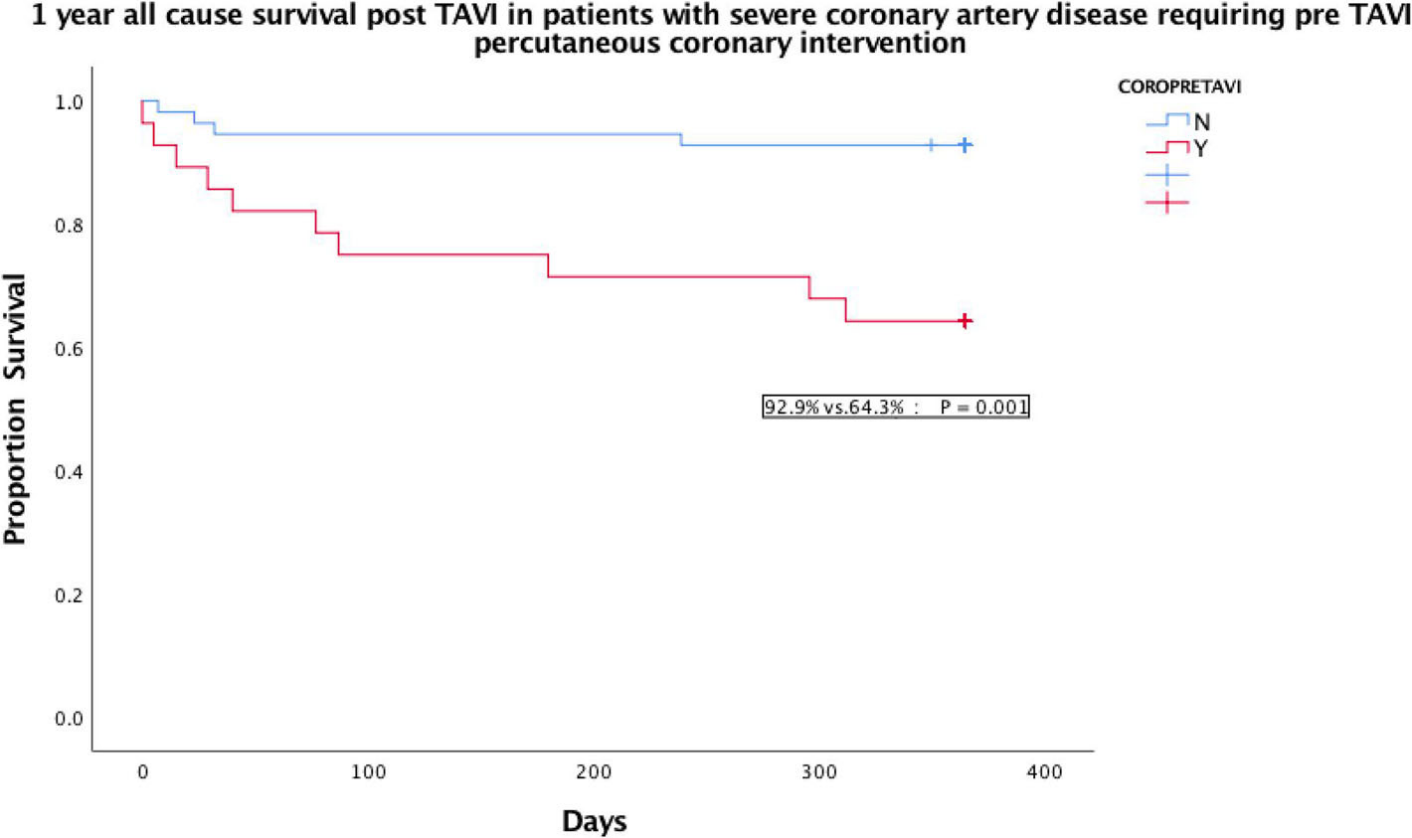
Kaplan Meier analysis of all cause survival post TAVI in patients with severe coronary artery disease requiring pre TAVI percutaneous coronary intervention

**Fig. 5:**
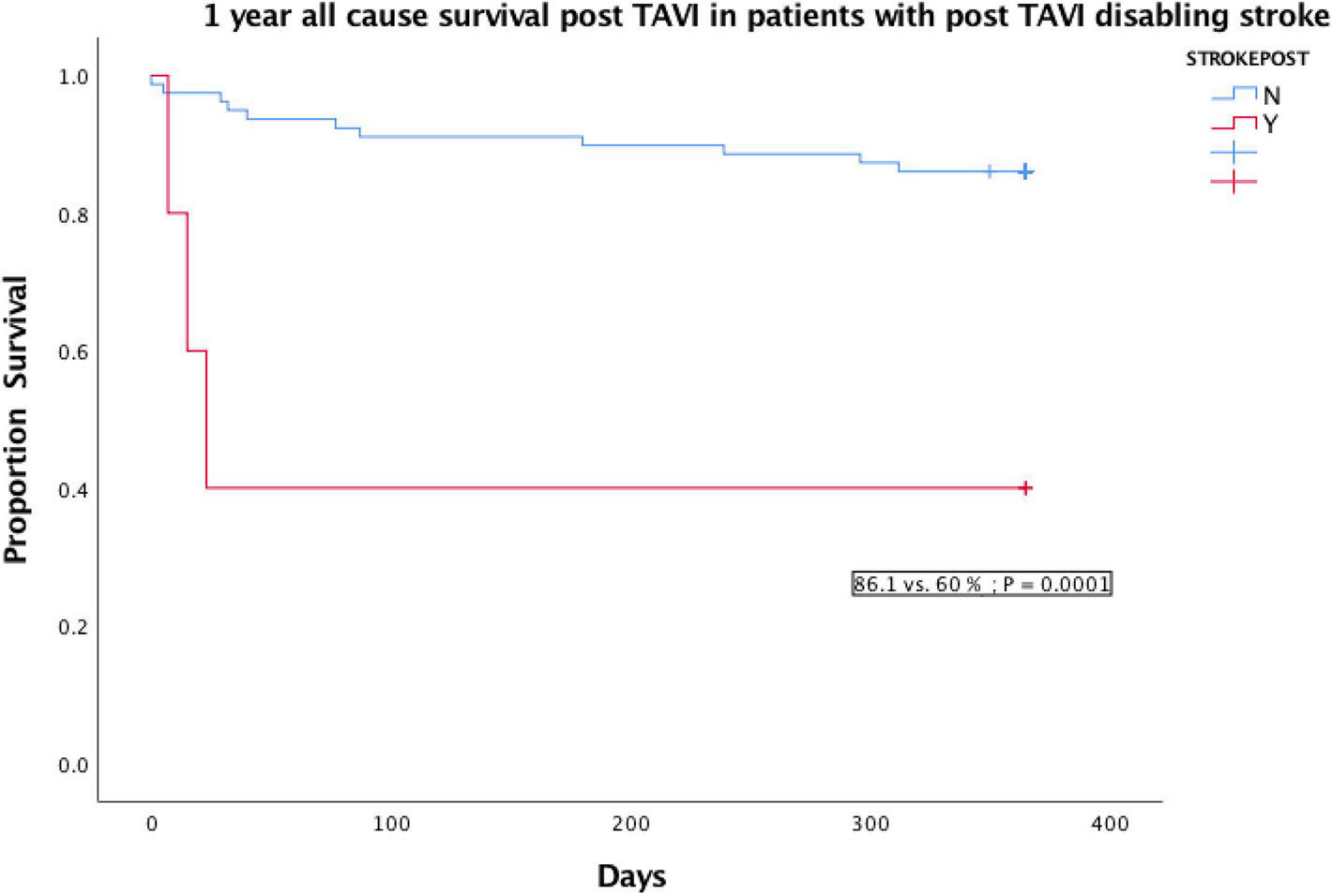
Kaplan Meier analysis all cause survival post TAVI in patients with post TAVI disabling stroke

Severe coronary artery disease requiring revascularisation (OR: 18.2 +/− 0.9; *P* = 0.002), pre TAVI atrial fibrillation (OR 8.6 +/− 0.9; *P* = 0.02) and post op disabling stoke (OR 32.6 +/− 1.35; *P* = 0.01) were found to be associated with 1 year all-cause mortality. On log rank test, severe coronary artery disease requiring percutaneous coronary intervention (PCI) and post TAVI disabling stroke significant impacted on 1 year all cause survival on Kaplan Meier analysis (Figs. 4 & 5). No correlation was identified between the need for PPM or moderate / severe PVL with 1 year all cause survival. The commonest indication for PPM implantation post TAVI was complete heart block / type III atrioventricular block (AVB III), followed by new onset left bundle branch block (LBBB) with symptoms and hemodynamic disturbance (Table 4). 12 out of 15 patients had their PPM implanted within 7 days post TAVI with 10/15 (66.7%) implanted between days 1–3 post TAVI. We analysed baseline ECG and pacemaker parameters during follow up and found that 6/15 (40%) patients with PPM implanted post TAVI were not considered to be pacemaker dependent on 1 year follow up. Amongst the 8 patients who had AVB III post TAVI with PPM, 3 / 8 (38%) were not dependent on pacemaker at 1 year. 5 out of the 12 patients (41.7%) with PPM implanted within 7 days post TAVI were not pacemaker dependent at 1 year.

**Table 4:** Post TAVI pacemaker implantation analysis

*Post TAVI PPM analysis (n = 15)*	
*Indications*	
*AVB type III*	8 /15 (53.3%)
*LBBB, HR < 40 bpm; symptoms*	3/15 (20%)
*Tachy-brady syndrome with rate < 40 bpm*	1/15 (6.7%)
*Slow Atrial fibrillation < 40 bpm; hypotension*	1/15 (6.7%)
*Slow Atrial fibrillation;* *long pause > 5 s*	1/15 (6.7%)
*Junctional/ Atrial fibrillation, with HR < 40 bpm*	1/15 (6.7%)
*DDDR*	5/15 (33.3%)
*VVIR*	10/15 (66.6%)
*Timing for PPM post TAVI*	
*Within 24 h*	4/15 (26.7%)
*D1-D3*	6/16 (37.5%)
*>D3 – D7*	5/15 (33.3%)
*More than 1 week*	3/15 (20%)
*VP & AP < 5% at 1 year of interrogation*	6/15 (40%)
- *AVB III as indication*	3/6
- *Tachy-brady*	1/6
- *Long pause*	1/6
- *Slow AF + LBBB + rate < 40*	1 /6
*Time for implant post TAVI for patients with VP/AP < 5% & ECG native > 60 bpm at Follow up*	
- *Within 24 h*	2/6
- *D1 to D3*	1/6
- *D3 to D7*	2/6
- *More than 1 week*	1/6

Presence of right bundle branch block (OR: 7.75 +/− 0.65; *P* = 0.002), NCC implantation depth (OR: 1.30 +/− 0.12; *P* = 0.03) and a NCC implantation depth/IMS ratio > 0.5 (11.3 +/− 1.12; P = 0.03) correlated with increased PPM implantation rates on univariate analysis. A longer IMS length (OR: 0.58 +/− 0.21; *P* = 0.01) was associated with a lower risk of PPM implantation. On multivariate analysis, pre TAVI RBBB (OR: 11.1 +/− 0.86: *P* = 0.005) and a deep NCC implantation depth (OR: 1.34 +/− 0.15; *P* = 0.048) were associated with increased risk for PPM implantation. Among the 57 patients with measured IMS length, a NCC implantation depth/IMS length > 0.5 (OR: 29.9 +/− 1.72; *P* = 0.05) was found to be related to higher PPM risks on multivariate analysis.

## Discussion

TAVI has firmly established itself as a mainstay treatment for patients with severe AS,and there is ample evidence supporting its safety and efficacy in patients at intermediate to high risk for SAVR. The PARTNER trials and the Corevalve trials have substantiated the role of TAVI in treatment of severe AS, and outcomes have been superior to SAVR. In our series, the device success rate was up to 88.6%, with a 30 day mortality of 5.7%. The 1 year all cause mortality rate was around 16.7%, and considering that most patients were octogenarians with high STS scores, we have demonstrated the safety and efficacy of TAVI in our centre. These survival outcomes are consistent with those reported in the high risk Corevalve study [8], and we expect outcomes to further improve as we move down the risk ladder to treat patients at low risk with low STS scores.

Nonetheless, we cannot discount the fact that most comparative trials between TAVI and SAVR included elderly patients and have excluded bicuspid aortic valves or valves with dense LVOT calcium. In addition, the issue of durability of TAVI valves and high pacemaker rates remain unanswered, and inevitably plays a significant role in deciding the use of TAVI in younger patients. As of today, SAVR remains the gold standard treatment for young patients at low risk of SAVR [9]. In our series, the PPM rate post TAVI was 17.6%. This rate was similar to the number from the TVT registry (16.6% for Evolut R) for self-expandable valves [10]. Multiple trials have reported varying rates of post TAVI PPM, with seemingly lower rates of PPM with balloon expandable valves versus self-expandable valves, but the rates still remain high compared to SAVR. Reported rates of PPM post TAVI range from 10 to 30%, which is unacceptably higher than the 3–6% of PPM in SAVR. Although randomized controlled trials have not demonsrated that the need for PPM negatively impacted survival comparing to SAVR, a recent 21 study meta-analysis on 42,927 patients have found that new onset LBBB and PPM implantation after TAVI were associated with an increased risk of all cause death and heart failure hospitalization at 1 year of follow up. The meta- analysis also concluded that new onset LBBB resulted in increased risk of cardiac death within 1 year post TAVI [5]. It is worth nothing that long term high quality studies are lacking concerning the effects new PPM implantation have on survival and cardiac events and function, and there is conflicting evidence suggesting otherwise. The REPRISE III trial, a randomized controlled trial between the LOTUS valve and Corevalve did not demonstrate worse clinical outcomes with new PPM [11].

Nonetheless, the lack of conclusive evidence relating new PPM to worse TAVI outcomes should not affect our resolve to further improve TAVI results in terms of reducing PPM rates. Numerous studies have looked into risk factors predisposing patients to a higher risk of PPM post TAVI and factors can be summarised as pre, intra and post procedural., Right bundle branch block is the most well established pre procedural risk factor for pacemaker implasntation post TAVI. For intraprocedural factors the use of self-expandable valves and a deeper implantation depth are associated with higher PPM risks. For post procedural factors, the occurrence of LBBB, AVB III and the need for rate control drugs have been reported to increase risks for PPM [12, 13]. In our study, we have identified RBBB and NCC depth as major predictors for post TAVI PPM. Jilaihawi et al. first reported the relationship between membranous septal length and the risk of PPM post TAVI. The PPM rate in their series with self-expandable valves was 9.7% and they found that by adopting an anatomically guided MIDAS (minimizing depth according to the membranous septum) approach to device implantation, aiming for an implantation depth less than the IMS length, could reduce need for PPM post TAVI [14]. In our study, we retrospectively measured the coaxial distance between the annulus and muscular ventricular septum on coronal cuts on CT scan and labelled the distance as infra-annular membranous septal (IMS) length. We found that the longer the IMS length, and the deeper the NCC implantation depth in relation to the IMS length predicted need for PPM. It is our postulation that by actively trying to position the TAVI valve as high as possible in relation to the IMS length can reduce the need for PPM post TAVI.

The REPRISE III trial demonstrated that pacemaker dependency was a dynamic phenomenon in which a majority of post TAVI patients with PPM implanted within 30 days post procedure were not pacemaker dependent. 20–40% changed dependency status at 1 year of follow-up. In our series, we analysed the pacing parameters upon follow up in all pacemaker patients and found that 6 out of 15 (40%) patients with new PPM were not pacemaker dependent based on our definition. Although there is no set criteria or definition for pacemaker non dependence. All 6 patients had a baseline ECG rate of 60 or above with % Ap/ Vp < 5% on pacemaker interrogation. Amongst the 12 patients who had their PPM implanted within 7 days post TAVI, 41.7% of them were not dependent on PPM at 1 year. Retrospectively, if we had waited for more than 7 days before implanting PPM in these patients we would have avoided 5 cases of PPM implantation, which would have reduced the rate of PPM to 11.7% from 17.6%. This finding has prompted us to rethink the timing of PPM implantation in TAVI patients, as it is common practice to defer PPM implantation to at least 7 days post SAVR. In our centre, more emphasis is put on early mobilization and discharge of patients post TAVI, and with a strong and readily available pacing team as support, there is a tendency to implant pacemakers more aggressively and early post TAVI. Perhaps it is time for us to adopt a more patient approach for post TAVI patients with new onset conduction disturbances before prematurely implanting PPM. In addition, more studies are needed to delve into factors that cause pacemaker dependence rather the risks of new onset conduction disturbance, as modifying factors that perpetuate pacemaker dependence may impact more on clinical practice.

The commonest conduction disturbance post TAVI is LBBB, and it is postulated to be related to pressure necrosis of the bundlecccnd NCC. It is not well understood why a repositionable, recapturable self-expandable valve has higher PPM rates than a self-centering balloon expandable valve. It is believed that the self expandable valve compresses the left ventricular outflow tract while the balloon expandable valve lands above that area and does not press on the his bundle as much. A recent large French registry has shown the SAPIEN 3 valve (Edwards Lifesciences Corporation, Irvine, California, USA) to have lower pacemaker rates than the Evolut R [15] . To date, there is no conclusive evidence showing superiority of BE valves over SE valves, but it appears preliminary data are strengthening the case of choosing a BE valve for patients at high risk of post TAVI PPM. Newer valve designs have emerged that rely less on LVOT/annulus radial expansion for anchoring and more on leaflet or supra-annular anchoring mechanisms [16, 17]. This was supposed to mitigate the risk of low valve implantation onto the LVOT causing conduction disturbances leading to PPM implantation, but so far more data are needed to substantiate this claim.

This study is retrospective in nature and has inherited intrinsic weaknesses such as recall bias, measurement bias and a lack of a comparison group. The population is relatively small and the findings relating to risk analysis are at best hypothesis generating. The measurement of the IMS length was liable to measurement error, as the quality of CT scans from different centres differed. The definition of pacemaker dependence could have been more stringent, but since the study was retrospective in nature, we were not able to proactively turn down the pacing rate to determine real dependence. This might potentially underestimate the rate of non-dependence.

## Conclusions

TAVI is safe and effective in patients with severe AS at intermediate to high risk for SAVR. Device success rates are high and outcomes are satisfactory. However, pacemaker rates remain high and there is a need to improve TAVI outcomes in order to fully realize the potential of TAVI in younger patients at low risk for SAVR. Pre TAVI RBBB and NCC implantation depth were important risk factors for post TAVI PPM. We have found an increased risk of PPM implantation post TAVI when the NCC implantation depth occupied more than 50% of the membranous septal length. We propose consideration of the IMS length during device implantation in patients at high risk of PPM implantation. More studies are needed to determine the optimal timing for PPM implantation and risk factors of PPM dependency post TAVI.

## Data Availability

The datasets used and/or analysed during the current study are available from the corresponding author on reasonable request.

## Abbreviations

AS: Aortic stenosis
AVB: Atrioventricular block
BE: Balloon expandable
IMS: Infra-annular membranous septum
LCC: Left coronary cusp
LVEF: Left ventricular ejection fraction
LVOT: Left ventricular outflow tract
MIDAS: Minimizing depth according to the membranous septum
mRS: Modified Rankin score
NCC: Non coronary cusp
PCI: Percutaneous coronary intervention
PPM: Permanent pacemaker
PVL: Paravalvular leak
RCC: Right coronary cusp
RBBB: Right bundle branch block
SAVR: Surgical aortic valve replacement
SE: Self expandable
TAVI: Transcatheter aortic valve implantation
